# Temporal experience modifies future thoughts: Manipulation of Libet’s W influences difficulty assessment during a decision-making task

**DOI:** 10.1371/journal.pone.0237680

**Published:** 2020-11-24

**Authors:** Eve A. Isham

**Affiliations:** 1 Department of Psychology, University of Arizona, Tucson, AZ, United States of America; 2 Center for Mind and Brain, University of California, Davis, CA, United States of America; Johns Hopkins University, UNITED STATES

## Abstract

Past studies have employed the subjective experience of decision time (Libet’s W) as an index of consciousness, marking the moment at which the agent first becomes aware of a decision. In the current study, we examined whether the temporal experience of W affects subsequent experience related to the action. Specifically, we tested whether W influenced the perception of difficulty in a decision-making task, hypothesizing that temporal awareness of W might influence the sense of difficulty. Consistent with our predictions, when W was perceived as early or late, participants subsequently rated the decision difficulty to be easy or difficult, respectively (Exp.1). Further investigation showed that perceived difficulty, however, did not influence W (Exp.2). Together, our findings suggest a unidirectional relationship such that W plays a role in the metacognition of difficulty evaluation. The results imply that subjective temporal experience of decision time modifies the consequential sense of difficulty.

## Introduction

A seminal study by Libet and colleagues examined the timing of decisions, questioning whether volition played a causal role in action output [1]. In the paradigm, participants performed a simple voluntary act (e.g., wrist flexion or a button press) and reported the time they felt the urge to act. This reported time, termed W, is often taken to signify the earliest moment in which one becomes aware of the moment of decision to execute an action. When evaluated in the context of a neural signature, termed the readiness potential [2], Libet observed that this brain activity preceded participant reports of W by approximately 300–800 ms [1]. Importantly, the latency of W relative to the timing of the readiness potential led to the interpretation that a conscious decision to act does not actually cause an action. In support of this view, Masicampo and Baumeister explained that the latency of W occurred too late in the event to be involved in the initiation of behaviors [3]. This led to the controversial interpretation that free will is simply an illusion [4] further prompting an unsettled debate of whether conscious experiences, at least as measured by the subjective assessment of W, have a function at all.

While Libet’s data led to the interpretation that consciousness has no function, Libet argued that in fact it did by pointing out that conscious will could have an influence on the action outcome in other circumstances [5]. He postulated that consciousness alerts the system to reconsider, and possibly cancel, unconsciously-initiated actions [5,6]. Libet proposed that such a cancelation process occurs during the temporal window between W and the voluntary motor response (e.g., a keypress). Although the presence of the readiness potential prior to judgments of action initiation might appear to argue for deterministic action, the ability to modify or cancel actions after a decision is reached could still serve as evidence for functional and purposeful consciousness.

A number of other theoretical proposals have suggested that consciousness serves the purpose of influencing and modifying future thoughts and behaviors. For instance, Masicampo and Baumeister proposed that conscious thought may have indirect effects on behavior, even if it does not directly control it [3,7]. For example, a study on mental simulations showed that experimentally enhancing the link between thought and action led to better performance on an exam in college students [8]. This perspective thus implies that conscious thoughts and mental content aid in the modification and planning of future behaviors [4,9].

In a recent study, we measured W and perception of difficulty during a decision-making task [10]. We found that judgments of W correlated with difficulty ratings such that later judgments of W corresponded with higher difficulty ratings and earlier judgments of W corresponded with lower difficulty ratings. In this previous study, we speculated that the temporal experience of W might influence the metacognition of difficulty assessment, although we did not directly test this issue. This observation gave rise to the current research question of whether the awareness of time W causally impacts successive thoughts and ideas.

If W can, in some instances, causally influence difficulty ratings, such findings might suggest that consciousness (as indexed by W) has a role in modifying subsequent thoughts and behaviors. A framework that explains this possibility is the Attended Intermediate-Level Representation theory (AIR) offered by Prinz [11]. According to AIR theory, consciousness serves the function of sending viewpoint specific information into the intermediate-level of processing (i.e., working memory). In turn, this allows for sequential planning and decisions throughout the course of action [12]. With respect to temporal consciousness in the Libet paradigm, it is possible that the timing of decision (i.e., W), which typically resides in the lower sensory level [e.g., 13], is brought forth to the intermediate, conscious level when the participants are asked to report W. As Prinz describes, at this intermediate level, information is represented in a specific viewpoint. Relating this to the results from Isham et al. [10], an awareness of early or late W could therefore have an influence on such a viewpoint, thereby influencing metacognition regarding difficulty evaluation.

In contrast to the above speculation, one could argue that the relationship between W and difficulty assessment observed in the Isham et al. study [10] is correlational rather than causal. Moreover, this relationship may be mediated by response time given it has been shown that metacognition of task performance (e.g., confidence ratings) depends partially on how fast one completes the task [14]. In Isham et al., participants had a sense of their response time (i.e., early vs late) [10]; in this manner, we cannot rule out the possible influence of response time on difficulty ratings from this earlier study. To successfully determine the influence of W on difficulty ratings, we must also rule out this simpler explanation of a mediating influence of response time on both W and difficulty ratings.

In the current study, we examined the effect of reported W (also referred to simply as “W”). We aimed to 1) demonstrate that reported W has an influence on difficulty ratings (Experiment 1); and 2) establish a causal relationship between reported W and difficulty ratings (Experiments 1 and 2). To meet Aim 1, we compared the effects of W reports and response time on difficulty ratings. We set up this assay experimentally such that in one condition, participants reported W, while in another condition, participants made no report of W at all (Experiment 1). A sense of how long it took to decide (i.e., response time), however, was present in both cases. If W were to influence difficulty judgment, independent of response time, then we might expect to see an effect of W only in the condition that required a W report. On the other hand, if response time were the primary contributor to the fluctuation in difficulty judgment, then the difficulty ratings would vary with response time despite the presence or absence of the W report.

Upon establishing that W reports have an influence on difficulty ratings in Experiment 1, we next examined whether the observed effect is a reflection of a causal relationship between W reports and difficulty ratings. We conducted Experiment 2 to determine whether W would vary with a difficulty rating manipulation. If the difficulty manipulation influenced the W report, then the relationship would be bidirectional and therefore not causal. On the other hand, if the manipulated difficulty had no influence on the W report, it would imply that W influences difficulty judgment, but not the reverse. Such a causal relationship, if established by our study, would imply that subjective temporal experience, as indexed by W, has an influential role in subsequent thoughts and ideas.

## Materials and methods

### Experiment 1

Participants performed a binary decision-making task by indicating whether they agreed or disagreed with different scenarios presented to them. Half of the participants reported W and the remaining participants did not. All participants rated difficulty of each decision made. The goal of Experiment 1 was to demonstrate that reported W had an influence on difficulty assessment in a decision making task. To achieve this goal, we biased the participants’ perception of W to be earlier or later via a tone manipulation procedure [15]. We anticipated the difficulty ratings to vary with W but not with response time.

#### Methods

*Participants*. Forty-eight participants were recruited from a pool of undergraduate student volunteers (38 females, 18–35 years old) at the University of California, Davis. All participants were fluent in English. The participants consented in writing to the study protocol which was approved by the Institutional Review Board of the University of California, Davis.

*Rationale and estimation of sample size*. The sample size estimation was computed based on our previous study [10], which observed the effect size of W associated with easy and difficult to be .31. Based on this, a power analysis revealed a sample size of 23 to be sufficient at alpha = .05, and power = 0.8 (G*Power 3, [16]) for the W group. The sample size was increased to 24 in the current study in anticipation of outliers.

For response time, we anticipated a small effect in the two comparisons of interest, one of which was a between-subjects variable (W Report vs No W Report Group). Therefore, we used an estimated effect size of .2. The estimated sample size was 40, suggesting that approximately 20 participants were needed in each of the between-subjects conditions. Given that 24 participants were needed in the W Report Group as previously described, we recruited 24 participants for the No W Report Group, resulting in 48 participants total for Experiment 1.

**Fig 1:**
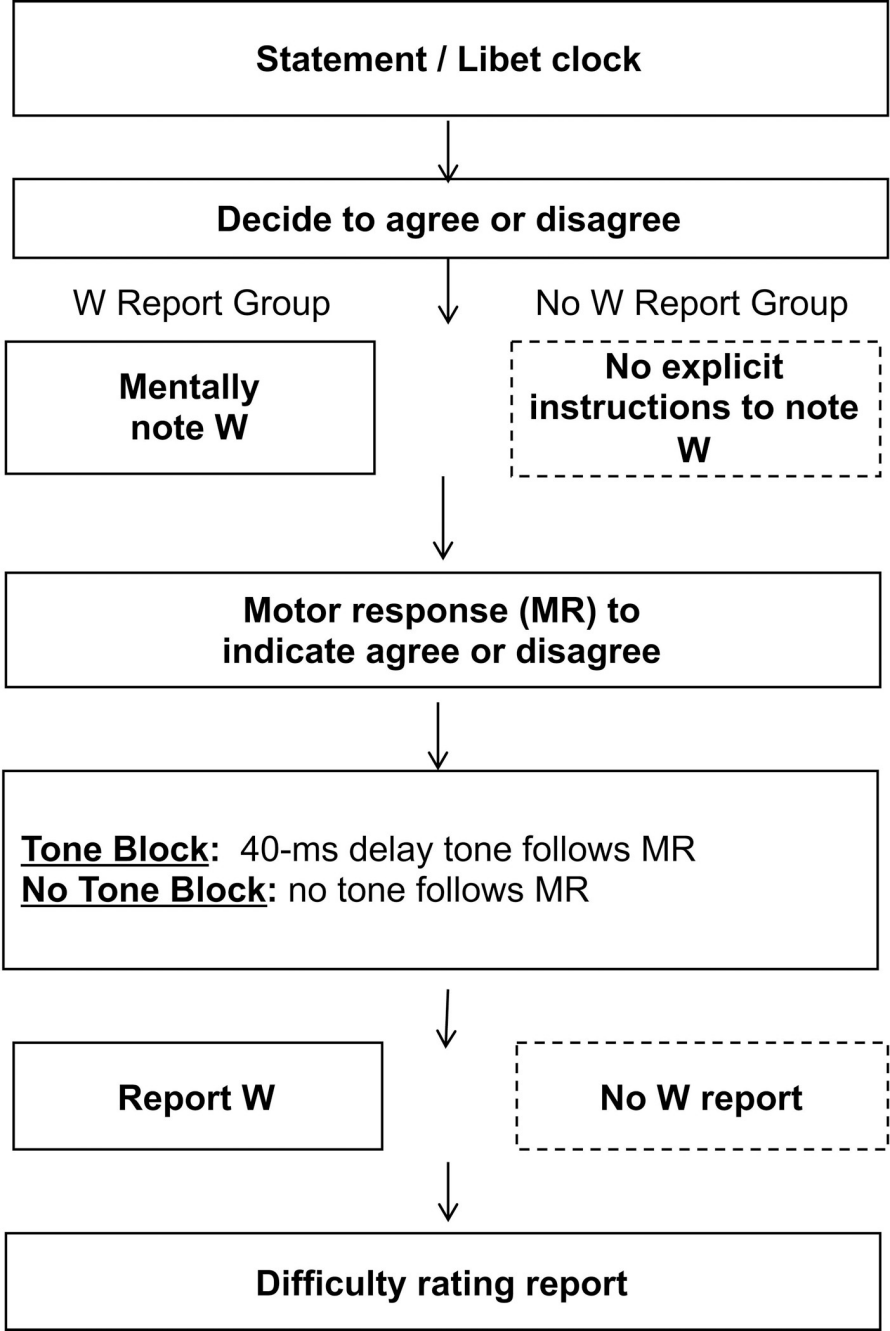
Experiment 1 procedure.

*Stimuli and Apparatus*. In Experiment 1, the participants listened to audio statements and were instructed to indicate whether they agreed or disagreed with each statement. Next they reported time W by reading the time from an analogue clock (like that used in Libet et al. [1]). Then, they rated the difficulty of the decision (summarized in Fig 1 and described in greater detail in the Procedure section below). In one experimental condition (No W Report condition), participants also answered a questionnaire at the conclusion of the experiment. To observe the influence of W on the difficulty ratings, we also manipulated the subjective report of W using a tone-manipulation procedure [15]. The statement stimuli, Libet clock, the tone used in the manipulation, and the post-interview questions are described below.

*Statement stimuli*: There were 111 statements total (adapted from Isham et al., 2017; see Supporting information for examples). Each statement is a member of a statement triad, sharing the same sentence structure but varying in the final few words at the end of the sentence. For example, the statement triad “I like red more than ___” consisted of three concluding words: “blue,” “green,” and “pink.” The statement triad “To save a village, it’s okay to sacrifice ___,” consisted of “a child,” “an animal,” and “food” as the final concluding words. In this manner, the 111 statements varied in degree of consequences within and across triads, rendering greater variability in difficulty ratings. Moreover, by having triad stimuli deviate from its members at the end of the sentence also helped minimize a premature decision-making process because the key deciding factor was not available until the end of the sentence.

The statements were recorded using a female speaker. The audio statements were edited using the software Audacity (Audacity, Boston, MA). The average statement length was 3037 ms (*SD* = 912). The statements were randomized and serially presented using Superlab (Cedrus Corporation, San Pedro, CA).

*Libet Clock*: While each statement was presented auditorily via the computer speakers, an analogue circular clock was simultaneously presented visually on the computer monitor, sharing the same onset time as the statement stimulus. The clock was 10.16 cm in diameter, positioned at the center of the screen, and was viewed from approximately 60 cm. Sixty tick marks were drawn along the circumference; a red dot moved along these tick marks, completing a rotation in 3 seconds. Each trial concluded at the end of the fourth clock rotation, giving each trial a duration of 12 seconds. The clock did not stop in response to the button press.

*Tone*: A critical component of the current experiment was to manipulate the perception of W to be later using a tone manipulation procedure [15]. The tone was created from a sine wave at 1000 Hz frequency and approximately 50 ms in duration (Audacity, Boston MA). As described in the Procedure section below, in half of the trials, the tone was delivered 40 ms after the decision was made (i.e., a keypress) while the remaining trials did not receive a tone.

Our prior work has shown that a tone, when presented after a brief delay, shifts time W to be later compared to when there is no tone [15]. From this earlier study, we used 5, 20, 40 and 60 ms delay tone (the 5 ms, rather than 0 ms, was the shortest that our technology was able to implement). Along with unpublished data from our lab, we observed W to be earliest when the tone was absent, with W shifting systematically to be later with the occurrence of the tone, demonstrating a form of temporal binding. Because a primary interest was to manipulate participants’ subjective W to be as early or as late as possible, we decided to use no tone and a 40 ms delay tone to maximize the effect. A 40 ms delay tone was more favorable than the 60 ms delay tone because temporal binding has been shown to decline around 60–80 ms [17].

*Post-experiment Interview*: Participants in the No W Report group received a two-item post-experiment questionnaire to ensure that the time component was not explicitly considered.

1. *“In terms of time*, *please elaborate on what you were thinking of when watching the clock*.*”* This question was used to assess whether participants explicitly thought of their decision time. No participants reported using the clock to time their decision and the clock did not prompt them to think about the timing of their decisions.
2. *“Please elaborate on your experience with the tone*.*”* This question was used to assess whether the participants were aware of the delay between timing of keypress and the tone presentation. No participants reported awareness of the delay manipulation.

*Procedure*. Fig 1 illustrates the step-by-step procedure in Experiment 1. The participants were randomly assigned to either the W Report or No W Report group. In both groups, the participants were instructed to listen to each statement carefully and entirely, and to make a decision to agree or disagree with the statement. While listening to each statement, the participants were also viewing the Libet clock. Those who were assigned to the W Report group were instructed to make a mental note of the position of the red dot on the clock when they felt they had reached a decision (i.e. “W”). It was emphasized that this was not the time in which they physically pressed the button but rather the earliest moment in which they became aware of having an inkling toward a decision. In this manner, our instructions mimicked those of Libet et al. [1] which asked the participants to report W as the moment in which they had the urge to act. It was done so in order to capture the earliest moment of awareness of the action. Participants in the No W Report group were also instructed to watch the clock but did not receive explicit instructions to note time W.

When a decision was reached, the participants indicated their decision by pressing the AGREE or DISAGREE key on the keyboard (we alternated between the A and the L keys). This motor response elicited a brief tone, delivered 40 ms after the keypress in half of the trials (Tone Block). In the remaining half of the trials (No Tone Block), no tone was delivered upon keypress. The order of Tone and No Tone blocks were randomized across participants.

Upon the motor response and tone delivery, when applicable, the participants in the W Report group verbally reported the time of their decision that was read from the clock, followed by a difficulty rating on a scale of 1 to 5, with 5 being the most difficult. Participants in the No W Report group reported only the difficulty rating and answered the two-item questionnaire about their temporal experience.

A total of 10 practice trials and 111 experimental trials were administered to each participant. Of these, half were randomly assigned to a Tone block and the remaining trials were randomly assigned to the No Tone block. The order of the Tone and No Tone blocks was counterbalanced. Note that the first of the two blocks always consisted of 56 trials; the additional trial served as an extra practice trial.

*Analysis*. The design of the experiment consisted of two independent variables: W Report Group (W is reported or W is not reported) and Tone Presence (Delayed Tone or No Tone). Three dependent variables were measured: W time, response time, and difficulty ratings. Response time was measured from the moment the statement ended to the time of keypress; greater response time indicates that the keypress was made later. W, as traditionally computed, was backward-referenced from the moment of action. A greater W value indicates that the perceived moment of decision was earlier and was further away from the time of action.

*Bayes Factor Analysis and False Discovery Rate Correction*: Bayes Factor analysis for all t-tests was included [18]. For results below the significance threshold (p < 0.05), we used a Bayes Factor, BF_10_, to indicate the degree of favorability toward the alternative hypothesis. For results that were not below the significance threshold, the Bayes Null factor, BF_01_, was used. The Cauchy prior r-scale was set at the default value of d = .707. The larger the Bayes Factor, the greater the evidence in support of the corresponding hypothesis being tested. To minimize the false discovery rate, we employed the Benjamini-Hochberg (BH) procedure for multiple comparison corrections [19].

#### Results

**Fig 2:**
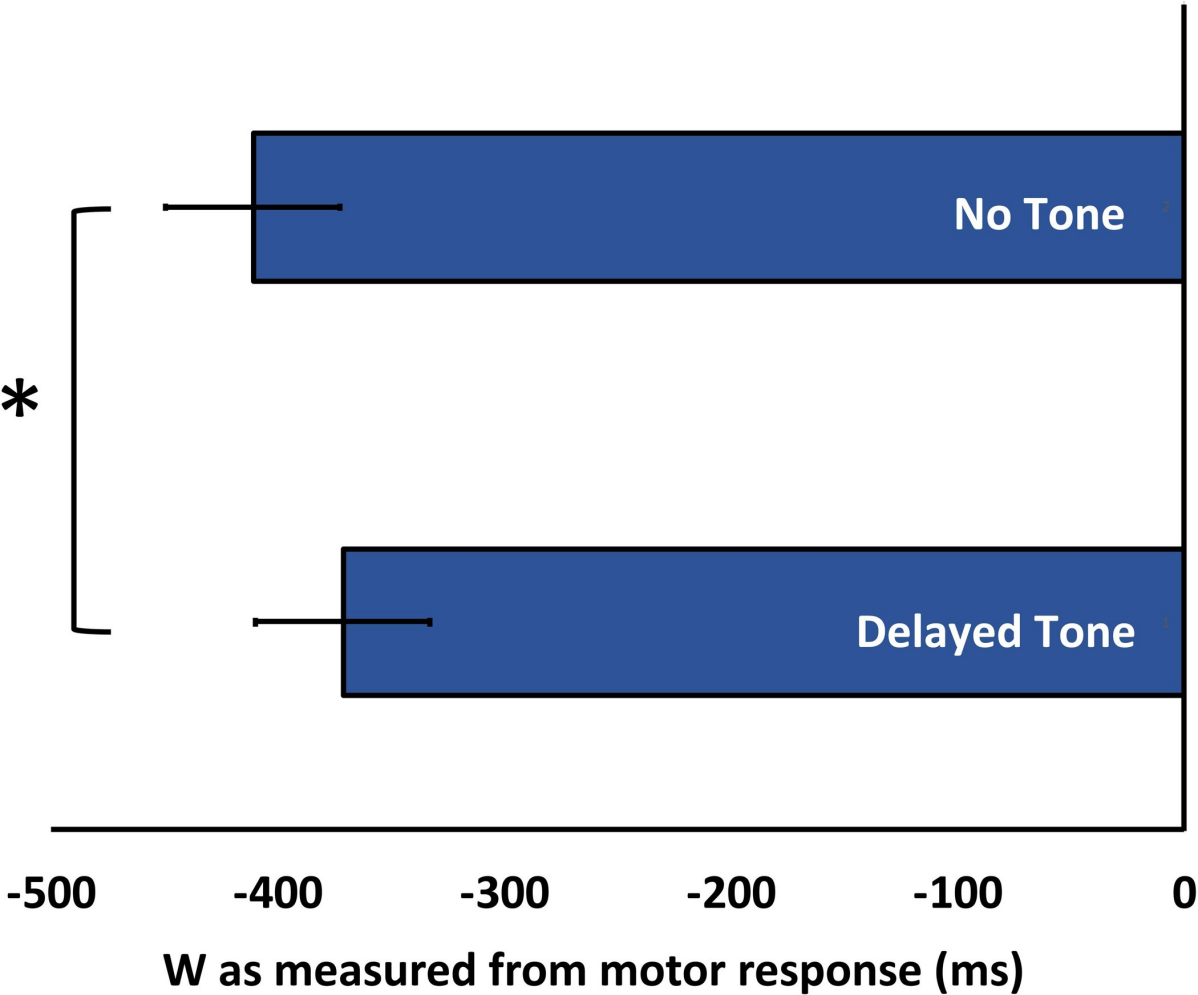
Reported W time. Reported decision time (W) as a function of Tone Presence. W was judged as later when the auditory feedback was delayed.

To determine that W had an influence on difficulty ratings, we must rule out a simpler explanation of a mediating influence of response time on both W and difficulty ratings. To do so, we must establish a scenario in which W and response time behave differently. According to our hypotheses, the presence or absence of the delayed post-action tone manipulation would bias the W report but not response time. The results are as follows:

*W time*. Time W was measured as the timing of decision relative to the timing of action. To illustrate the effect of the tone manipulation on W, the W reports were subjected to a paired sample t-test comparing the Delayed Tone and No Tone conditions. Replicating our previous work [15], we observed the tone manipulation successfully altered the perceived time of decision such that when a delayed tone was given, the perceived time of decision (*M* = 371.31 ms before keypress, *SE* = 38.60) was later than when no tone was given (*M* = 410.90 ms before keypress, *SE* = 38.49), *t*(23) = 2.47, *p* = .021, corrected for multiple comparisons (BH *p-*value = .042), BF_10_ = 2.58. The mean W times are represented in Fig 2.

*Response time*. Of the 24 participants recruited for the No W Report group, two participants’ response time values exceeded two standard deviations of the group mean. Therefore, only 22 participants in the No W Report group were included in response time analyses.

**Fig 3:**
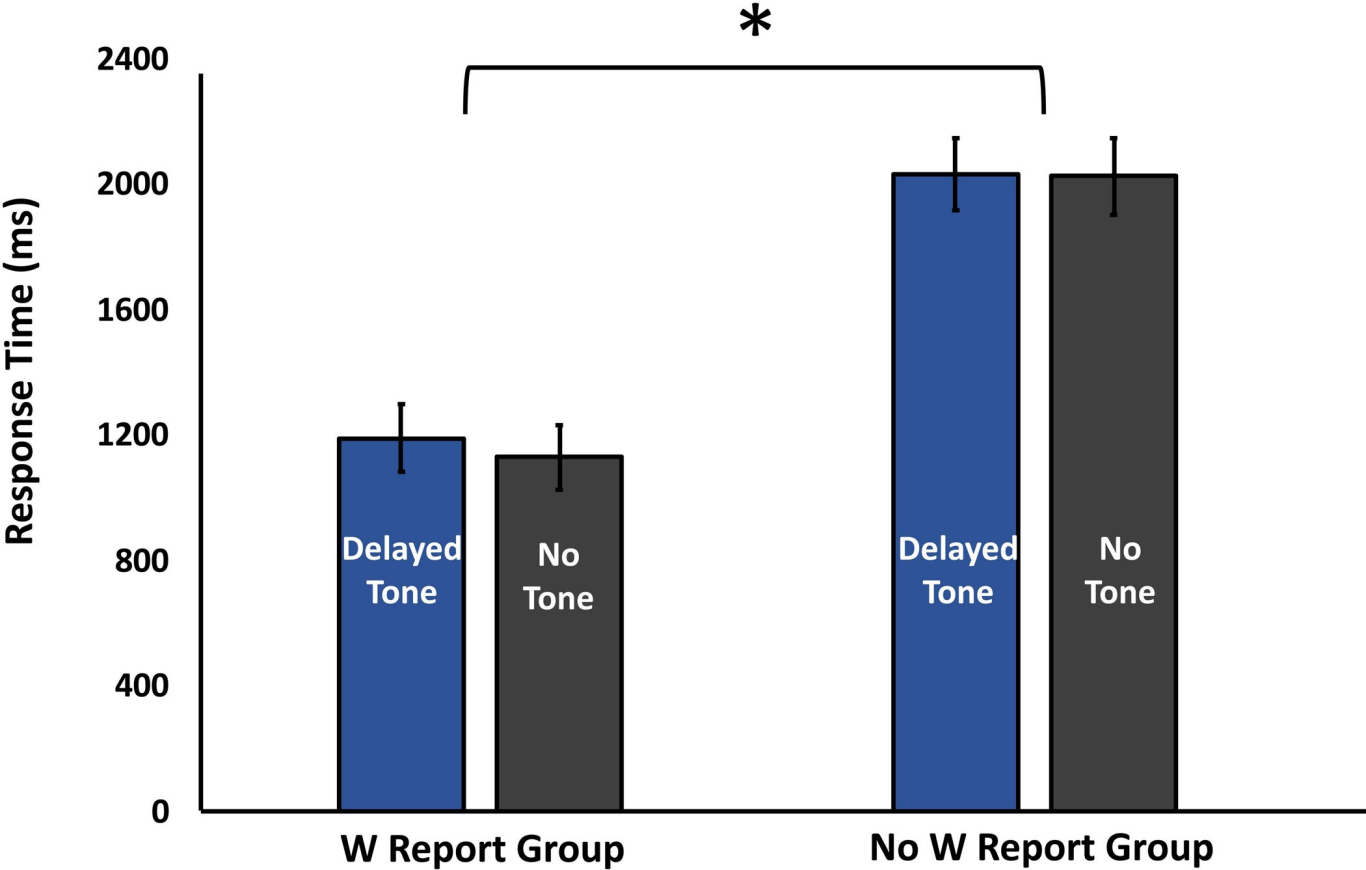
Response time. Response time as a function of Tone Presence and W Report Group. Response time did not vary with the tone manipulation. However, longer response time was observed in the No W Report group.

In contrast to W, we did not anticipate the tone manipulation to have an effect on response time. The response time data were subjected to a 2 Tone Presence x 2 W Report Group mixed ANOVA. Mean response time data are shown in Fig 3. As predicted, the results revealed no main effect of Tone Presence, *F*(1,44) = .606, *p* = .441, η^2^ = .014. Further examination of the W Report group revealed no difference in the mean response time of the Delayed Tone Condition (*M* = 1193.63 ms, *SE* = 104.36) and the No Tone condition (*M* = 1133.74, *SE* = 98.32), *t*(23) = 1.118, *p* = .275, BH *p-value =* .344, BF_01_ = 2.7. Similarly, examination of the No W Report group also revealed no difference in response time between the Tone (*M* = 2034.43, *SE* = 115.47) and No Tone condition (*M* = 2028.90, *SE* = 121.16), *t*(21) = .084, *p* = .934, BH *p-value* = .933, BF_01_ = 4.5. In addition, there was no interaction effect between the Tone Presence and W Report Group, *F*(1,44) = .418, *p* = .521, η^2^ = .009, suggesting that the tone manipulation did not have an impact on response time. These response time patterns differ from those for W and thus provide a condition in which response time and W are dissociated. In other words, while the presence of the tone had an effect on participant reports of W, it did not impact response time.

Interestingly, there was a main effect of W Report Group on response time. The analysis revealed that response time was shorter in the W Report group (*M* = 1163.68, *SE* = 103.23) compared to the No W Report group (*M* = 2031.67, *SE* = 107.82), *F*(1,44) = 33.814, *p* < .001, |^2^ = .435, BH *p-value* < .001, BF_10_ = 19480.61. This finding is discussed below.

**Fig 4:**
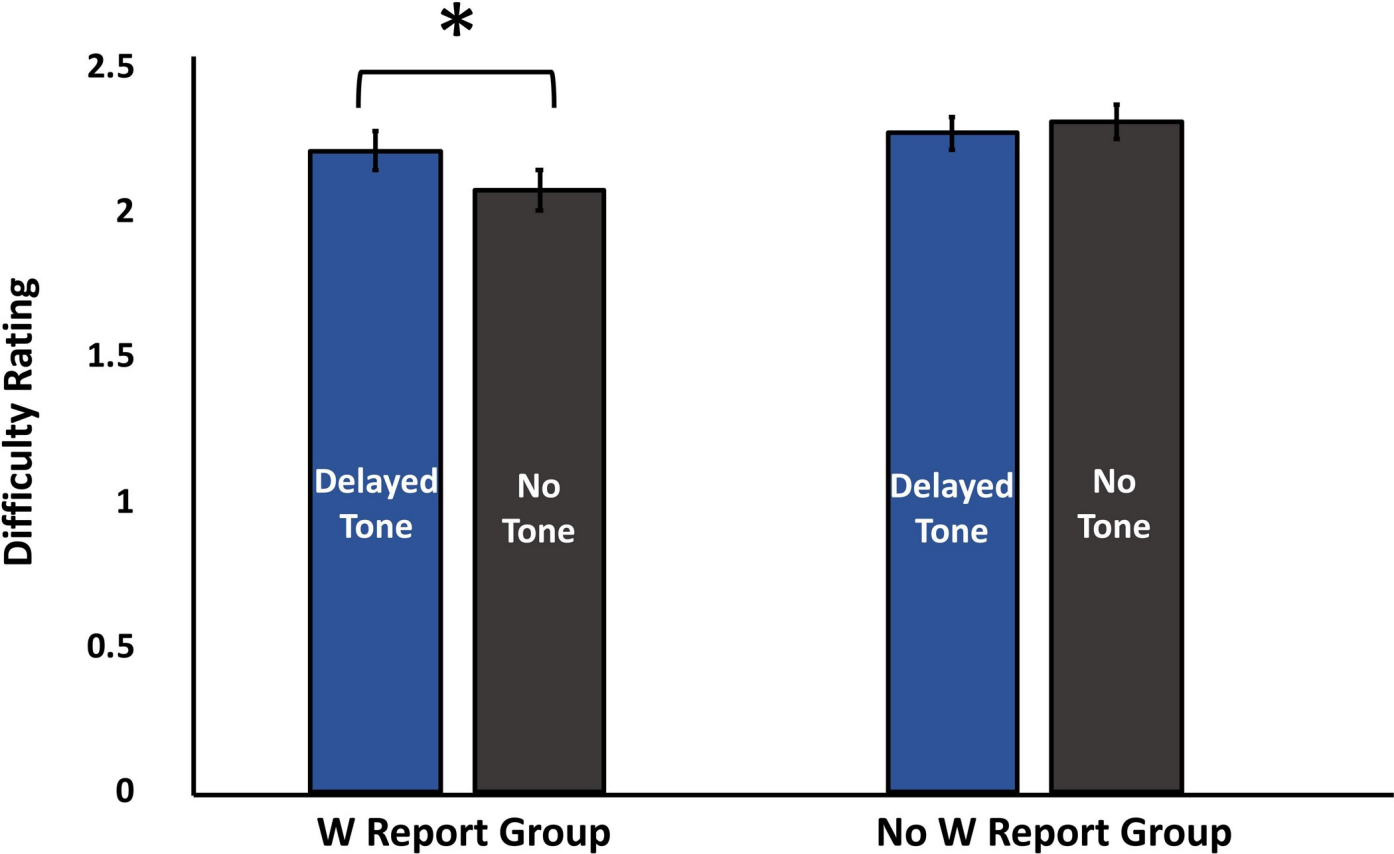
Difficulty rating. Difficulty rating as a function of Tone Presence and W Report Group. The tone manipulation affected the difficulty assessment such that the tone elicited a higher difficult rating. The effect of the tone manipulation was observed in the W Report Group but not in the No W Report Group.

*Difficulty rating*. The two previous analyses showed that W varied with the presence of the tone while response time did not. We next examined whether difficulty rating behaved in the same manner as W with respect to the presence of the tone. The difficulty rating was subjected to a 2 Tone Presence x 2 W Report Group mixed ANOVA. The analysis revealed an interaction effect between Tone Presence and W Report Group, *F*(1,46) = 5.105, *p* = .029, η^2^ = .100. Pairwise comparisons further showed that the interaction effect was attributed to the influence of Tone Presence in the W Report Group such that the difficulty rating was higher when the delayed tone was present (*M* = 2.21, *SE* = .06) than when the tone was absent (*M* = 2.08, *SE* = .07), *t*(23) = 2.45, *p* = .023, BH *p*-value = .042, BF_10_ = 2.5. The difficulty rating scores in the Delayed Tone and No Tone conditions, however, were not statistically different in the No W Report Group (*M* = 2.27, *SE* = .06 in the Delayed Tone condition; *M* = 2.31, *SE* = .07 in the No Tone Condition), *t*(23) = 0.71, *p* = .483, BH *p*-value = .511, BF_01_ = 3.7. The difficulty rating data are represented in Fig 4.

The interaction effect observed in this analysis suggests that when time W is explicitly accessed (i.e., W Report Group), the difficulty ratings are affected by the tone manipulation. However, the difficulty ratings are unaffected in the absence of W report. This key finding suggests that the temporal experience of W plays an important role in difficulty judgments.

In addition to the interaction effect, there was a marginal main effect of the W Report. The ANOVA revealed that the W Report group’s mean difficulty rating (*M* = 2.15, *SE* = .06) was lower than the mean rating given by the No W Report group (*M* = 2.29, *SE* = .06), *F*(1,46) = 3.406, *p* = .071, η^2^ = .069. However, this did not pass the Benjamini-Hochberg test (BH *p*-value = .107). Since the W Report variable was a between-subjects variable, it might be the case that there was greater variability in the sampled population, making the results inconclusive.

#### Discussion

In this experiment, we have established a situation in which W and response time are dissociated: W, but not response time, varies with a delayed auditory feedback. The observed main effect of the tone manipulation on W was expected and served as a manipulation check. The absence of a main effect of the tone manipulation on response time was also expected since the tone came after the motor response and therefore should not have an influence on the timing of the motor execution.

Our key finding is the effect of the tone manipulation on difficulty assessment. We have demonstrated that when the tone was presented, difficulty ratings were higher than when the tone was absent. Importantly, this effect was observed only in the W Report group, but not in the No W Report group. An interpretation of this key interaction effect is that the tone manipulation influenced the subjective W in the W Report group, and in turn, W influenced difficulty ratings. In the absence of the W report, the tone manipulation was ineffective. This interpretation therefore implies that the temporal experience of W plays a role in subsequent metacognitive process of difficulty assessment.

The post-interview results observed in the No W Report group further emphasize the influence of W as a conscious subjective experience. The interview provided additional qualitative data that participants in the No W Report group were not monitoring their temporal experience and did not explicitly incorporate timing information. As a result of inattentiveness to timing of decision, difficulty ratings did not vary with the tone in the No W Report group. Aligned with the AIR theory [12], this observation is consistent with the perspective that timing of decision may possibly reside at a lower level of consciousness [13]. When instructed to explicitly attend to it, the timing of decision then emerges to a more aware, intermediate level of consciousness. Upon entering this stage, the timing information consequentially affects the difficulty assessment.

A concern with the results is that the change in difficulty rating might not have been related to the timing of decision, but instead is an artifact related to an interruption in the performance due to the presence of the tone. This concern can be addressed by the interaction effect observed in the difficulty rating analysis. Recall that the tone manipulation was administered to both the W Report and the No W Report groups. The effect of the tone manipulation on ratings, however, was observed only when the W report was solicited. Therefore, this suggests that interference of the tone, if any, was not the primary contribution of the tone. Rather, it was the W experience that played a significant role in difficulty assessment.

It is also important to note the main effect of the W Report on response time; shorter response time was observed in the W Report group compared to the No W Report group. One speculation is that the participants in the W Report group might have been more attentionally engaged in the overall task given they were explicitly asked to identify and remember W. Given this focus, it could have led to a shorter response time. If this speculation were empirically supported, it would add to the claim that awareness of the reported time of decision has consequences in related thoughts and action.

The results thus far provide evidence for a close relationship between subjective timing of decision (W) and difficulty ratings. However, it is unclear if the relationship is unidirectional such that W affects difficulty rating, or whether anticipated difficulty level has an impact on W, as it could be speculated that W is inferred from the ratings. We examined these concerns in Experiment 2.

### Experiment 2

In Experiment 2, we investigated whether prospective knowledge or perception of difficulty influences the perceived time W. To manipulate the perception of difficulty, two sets of stimuli of medium-rated difficulty were assigned to either an EASY or DIFFICULT set as detailed below. W reports for these “manipulated easy” and “manipulated difficult” stimuli were compared. If the manipulation influenced the W report when evaluating these stimuli, it would imply that W and difficulty judgment covary. On the other hand, if W did not vary with the difficulty manipulation, it would suggest that the relationship observed in Experiment 1 is unidirectional.

#### Methods

*Participants*. Twenty-four participants (17 females, 18–50 years old) were recruited from a pool of undergraduate student volunteers at the University of California, Davis. The stimuli were drawn based on survey results given by separate, independent participants (67 participants; 44 females, 18–32 years old) also recruited from a pool of undergraduate volunteers at the University of California, Davis. All participants were fluent in English. All participants consented in writing to the study protocol which followed the guidelines approved by the Institutional Review Board of the University of California, Davis.

*Rationale and estimation of sample size*. Consistent with Experiment 1, we recruited 24 participants.

*Survey and Stimuli*. A separate group of participants (N = 67) rated the difficulty of making an Agree/Disagree judgment for each of 111 statements using a Likert scale of 1 to 5 (5 being most difficult). The 15 easiest and the 15 most difficult statements were assigned to the “True Easy” and “True Difficult” conditions, respectively. We next identified 30 statements that were rated of medium difficulty (ranked between 41 to 70 out of the 111 statements). These 30 statements were further divided into two sets of stimuli. To ensure the overall equality of the two sets, statements were assigned to each set according to their ranking order being odd or even; odd ranking numbers were assigned to Set A, and even ranking numbers were assigned to Set B. The 15 True Easy statements and the 15 statements from Set A (Manipulated Easy) made up the EASY condition. The set of 15 True Difficult statements and the 15 statements from Set B (Manipulated Difficult) made up the DIFFICULT condition. Assignment of the two medium sets to the EASY and DIFFICULT conditions was counterbalanced, and the 30 stimuli within each condition were randomized across participants. All stimuli for Experiment 2 can be found in Appendix 1.

*Procedure*. Participants were presented with a practice session (10 trials) followed by blocks of either 30 EASY or DIFFICULT statements, and then the remaining block of 30 DIFFICULT or EASY statements. The presentation order of EASY and DIFFICULT blocks was counterbalanced. As in Experiment 1, participants were asked to make a decision about each statement and to report W and the difficulty rating. To manipulate the perception of difficulty, we implemented the following scripts at the beginning of the EASY and DIFFICULT experimental blocks.

EASY: *From a survey of 100 people*, *these statements were considered to be easier to evaluate*. *On a scale of 1 to 5*, *1 being very easy and 5 being difficult*, *the average rating was 1*.*72*. *We are curious how you would rate them*.

DIFFICULT: *From a survey of 100* people, *these statements were considered to be more difficult to evaluate*. *On a scale of 1 to 5*, *1 being very easy and 5 being difficult*, *the average rating was 4*.*36*. *We are curious how you would rate them*.

Aside from these additional scripts, the remaining procedure and instructions were similar to the W Report (tone absent) condition in Experiment 1. Participants verbally reported W and provided a difficulty rating on a scale of 1 to 5 (5 being most difficult).

#### Results

Three dependent variables, response time, difficulty rating, and W were subjected to separate within subject one-way ANOVAs and post-hoc pairwise comparisons.

**Fig 5:**
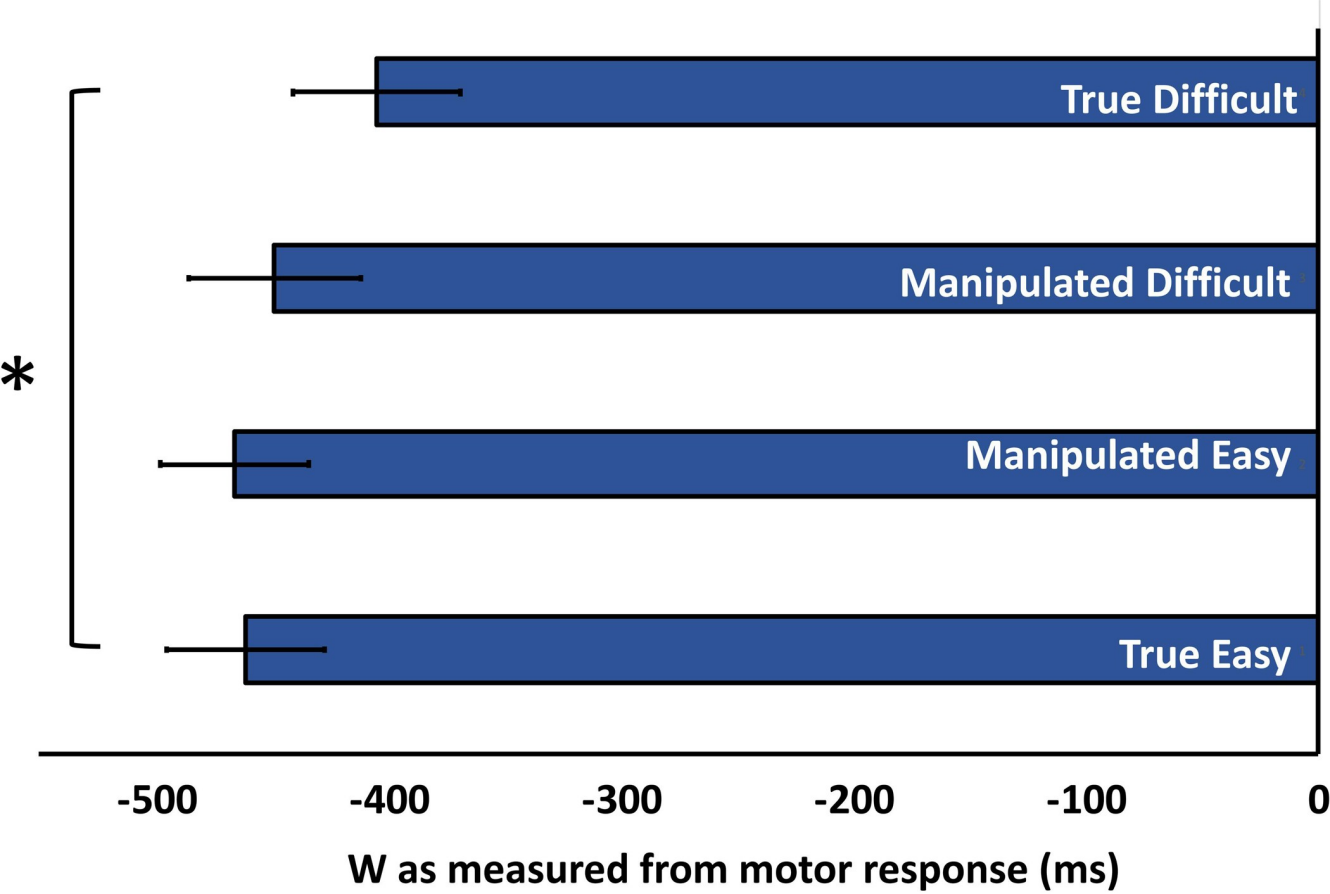
Difficulty rating as a function of the difficulty manipulation. Medium-rated statement stimuli were placed in either the Manipulated Easy and Manipulated Difficult conditions. Lower difficulty ratings were given to these stimuli when placed in Manipulated Easy condition compared to the Manipulated Difficult condition.

*Difficulty rating*. The primary purpose of this analysis was to demonstrate the effectiveness of the difficulty manipulation. We observed a significant difference across the four categories of stimuli, *F*(3, 69) = 63.074, *p* < .001, η^2^ = .733. Post-hoc pairwise comparisons showed that the True Easy condition was judged to be the easiest (*M* = 1.43, *SE* = .08), and the perceived difficulty significantly increased with Manipulated Easy (*M* = 1.91, *SE* = .13), Manipulated Difficult (*M* = 2.40, *SE* = .10), and True Difficult (*M* = 2.93, *SE* = .13), *p* < .001. Importantly, the difference in the ratings between the Manipulated Easy trials and the Manipulated Difficult trials was .48, and this was statistically significant, *t*(23) = 4.213, *p* < .001, BH *p-value* < .001, BF_10_ = 92.96. The results thus served as a check that the manipulation of difficulty was effective. The difficulty rating data are depicted in Fig 5. We next examined whether W varied according to the manipulated difficulty.

**Fig 6:**
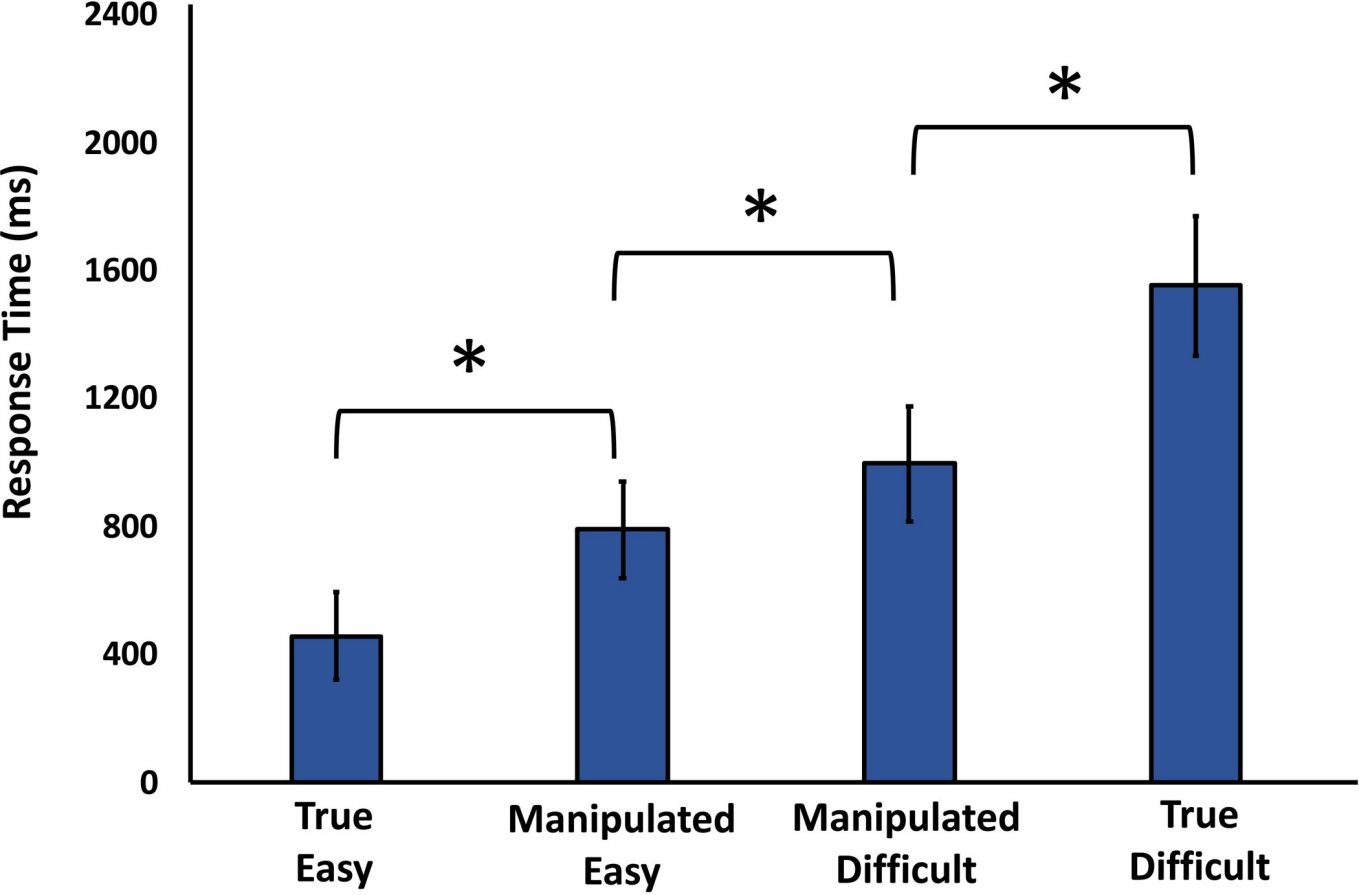
Reported decision time (W) as a function of difficulty manipulation. Medium-rated stimuli were placed in the Manipulated Easy and Manipulated Difficult conditions. W reports were not statistically different between these conditions.

*W*. A one-way ANOVA showed a significant difference across the four categories of stimuli, *F*(3,69) = 4.871, *p* = .004, η^2^ = .175. Post-hoc pairwise comparisons further showed a significant difference in the W reports between the True Easy (*M* = 462.14 before keypress, *SE* = 34.18) and the True Difficult (*M* = 405.57 before keypress, *SE* = 36.27), *t*(23) = 2.504, *p* = .020, BH *p-*value = .024, BF_10_ = 2.74. Consistent with our *a priori* hypothesis, this finding replicated our prior report that an easy decision is associated with an earlier W and a difficult decision is associated with a later W (Isham et al., 2017). The critical observation though is the lack of statistical difference in the W reports between the Manipulated Easy (*M* = 466.86 before keypress, *SE* = 31.83) and Manipulated Difficult conditions (*M* = 449.59 before keypress, *SE* = 36.88), *t*(23) = 1.006, *p* = .325, BH *p-value* = .366, BF_01_ = 2.96. This result suggests that difficulty judgments do not influence W. The W data are depicted in Fig 6.

**Fig 7:**
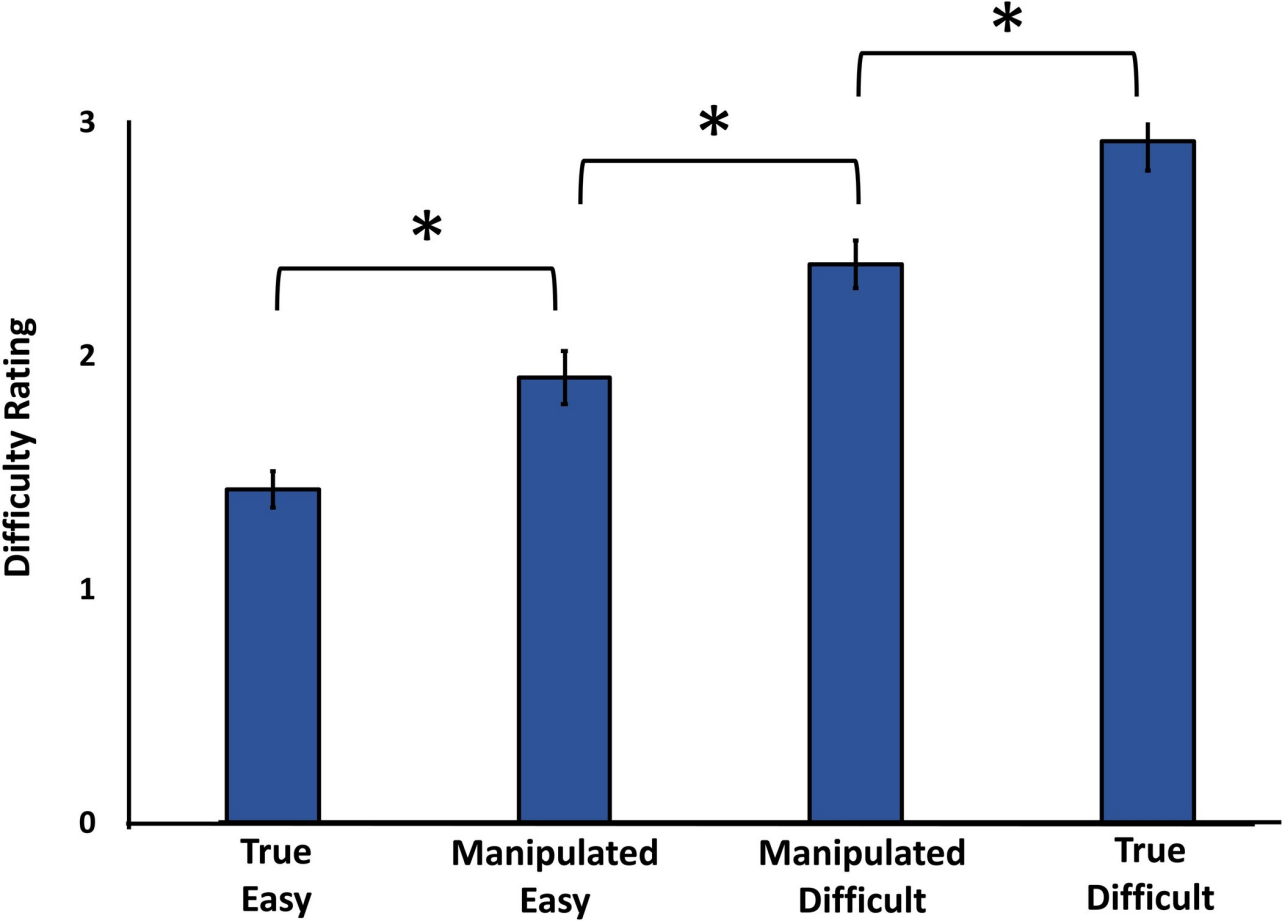
Response time as a function of difficulty manipulation. Medium-rated stimuli were placed in the Manipulated Easy and Manipulated Difficult conditions. Response time in the Manipulated Easy condition was shorter than in the Manipulated Difficult.

*Response time*. The response time was subjected to a one-way ANOVA. There was a main effect of the different types of trials, *F*(3,69) = 39.105, *p* < .001, η^2^ = .630. Post-hoc pairwise comparisons showed that the mean response time varied across the four conditions tested: True Easy condition (*M* = 461.08, *SE* = 136.18), Manipulated Easy (*M* = 793.50, *SE* = 150.97), the Manipulated Difficult (*M* = 999.40, *SE* = 180.92), and the True Difficult (*M* = 1554.94, *SE* = 218.50). As expected, there was a significant difference in response time between the True Easy and True Difficult conditions, *t*(23) = 7.768, *p* < .001, BH *p-value* < .001, BF_10_ = 207357.3. Furthermore, there was a significant difference of 205.90 ms between the mean response time of the Manipulated Easy and Manipulated Difficult, *t*(23) = 2.585, *p =* .017, BH *p-value* = .021, BF_10_ = 3.18. This suggests the possibility that difficulty judgment and response time are related. However, further investigation is needed to examine whether this is causal and whether difficulty evaluation could influence the pre-planning of how much time to spend on a decision. The response time data are depicted in Fig 7.

#### Discussion

The purpose of Experiment 2 was to examine whether biases in the perception of difficulty influence the estimates of W or response time. We successfully biased the belief that the target stimuli, which otherwise were equated, were either easy or difficult to evaluate. Despite the effectiveness of the manipulation, the manipulated stimuli did not influence W. In conjunction with the results from Experiment 1, this lack of effect on W supports the perspective that the relationship between W and difficulty is unidirectional such that W influences the perceived degree of difficulty, but not the reverse.

With respect to response time, the results suggest the possibility that response time covaries with perceived difficulty. It might be the case that a trial, when perceived as easy, encourages participants to spend less time making the decision; and a trial, when perceived to be difficult, encourages participants to spend more time deliberating before reaching a decision. This corroborates a previous observation that perceived difficulty influences response time [e.g.,^20^]; however, the result is inconsistent with the view that response time contributes to the experience of task difficulty [e.g., 21]. Thus, further examination specific to the response time manipulation is recommended.

## General discussion

Brass and Haggard propose the temporal concept of “When” as one of the three key components underlying decision making [22]. The current study investigates whether the subjective temporal experience of decision (W) modifies the metacognition of difficulty judgments. In two experiments, we observed that the temporal experience of decision had the priority over actual response time in modifying judgments regarding decision difficulty. That is, in Experiment 1, we observed that the perception of difficulty varied with the perception of when a decision was made (time W). When participants experienced delayed auditory feedback, they judged W to be later and reported their decisions to be more difficult compared to when there was no tone and they judged W to be earlier. On the other hand, when we manipulated perceived difficulty, participants did not change their reported time W (Experiment 2).

**Fig 8:**
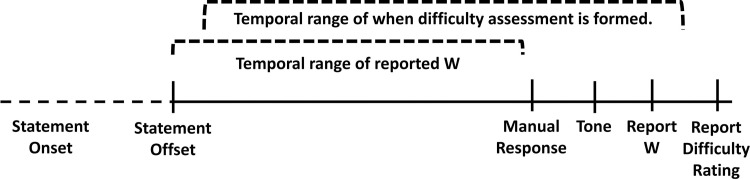
Timing for the formation of W and difficulty assessment. The timing of reported W and a participant’s assessment of difficulty are theorized to occur at any time after the offset of the stimuli. One perspective is that difficulty assessment occurs after the subjective temporal experience of decisional inkling as marked by the reported W. Note: Reported W may reflect a combination of veridical W and a bias facilitated by the tone manipulation.

A possible explanation to this observation is built on the notion that decision making operates within a hierarchical framework. This framework begins with the thought of the decision, whose timing is indexed by W, that precedes the evaluation of decisional difficulty (difficulty rating). On this basis, we propose that the W report is flankered by the stimulus offset and the time of the manual response. Difficulty assessment is occurs any time after W. That is, difficulty assessment may occur before the committed manual button press or after (Fig 8).

The idea of hierarchical order of events is further supported by the findings from Experiment 2, which showed that the manipulated difficulty impacted response time. This effect may be related to planning; when we expect a decision to be difficult, we may plan to spend longer time on the problem. Further investigation is necessary to verify the possibility of this causal relationship. If supported, this would challenge the perspective that response time influences difficulty ratings [e.g., 21].

### Mechanisms related to the function of consciousness

We propose that the observed effect of W on the difficulty ratings leads to the interpretation that consciousness serves to modify future thoughts and behaviors. While we believe the current findings support this perspective, the mechanistic component related to our findings remains unresolved. As discussed earlier, our results may map on to the framework of the AIR theory offered by Prinz [11]. According to this theory, consciousness serves the function of sending viewpoint specific information from a lower sensory level to an intermediate level of processing. Even so, it is unclear what facilitates the migration from one level to the next.

A memory-based theory of consciousness may help explain how this is possible. Evidence summarized by Kanai et al. [23] suggests the possibility that consciousness is involved in maintaining sensory information in short term memory. Such an idea connects with the findings from our current study. In our paradigm, participants maintained and integrated sensory information from visual (clock reading), tactile (button press) and auditory (feedback tone) modalities to produce a W report. Linking this to the AIR theory, we speculate that consciousness’ role in short term memory maintenance helps keep the integrated sensory information at the intermediate level or higher. In so doing, it is possible that the intermediate level is also where meta-cognition about the timing of a decision is formed or updated. Generally speaking, memory is flexible, fluid, and is likely updated when new information is available; e.g., Loftus and Palmer’s landmark study showed how additional information can alter one’s memory of a witnessed car accident [24]. We believe this may generalize to memory for the timing of decision; when presented with a tone, participant’s perception of W is updated. Subsequently, when prompted to evaluate difficulty, we infer from this most current version of W to produce the difficulty report.

Another key finding from the current study is that the effect of W on difficulty judgment was only observed when W was explicitly attended. This observation, and in conjunction with the fact that the experimental task involved non-perceptual, subjective statement stimuli, implies that the reported W was a more deliberate and of a higher-level conscious thought process. This conclusion concurs with a previous study whose drift diffusion approach illustrated that decision time was a deliberative process, rather than perceptual [25]. Upon this assumption, the current findings have the potential of addressing the extent in which conscious and non-conscious decision time play a role in the metacognition of difficulty judgment.

Relatedly, one speculation resonates with the idea that the effect of W on difficulty ratings operates as a low-level priming effect. That is, an early W could prime an easy rating and a late W could prime a higher difficulty rating. If additional investigation demonstrated support for such low-level priming effect, it would invite additional questions surrounding W and difficulty ratings. Particularly, why would low-level priming effect apply only in the context in which the W unidirectionally affected ratings (i.e., Experiment 1), but presumably would not apply in the reverse scenario (i.e., difficulty judgment did not affect W judgment, Experiment 2). Extended investigation on this context-dependent intersection between metacognition and low-level cognition thus could expand further the relationship between consciousness and decision making.

In addition to the difficulty rating data, analysis of the response time difference between the W and No W Report group also supports the perspective that consciousness has an influence on future thoughts and action. From Experiment 1, we observed that the participants in the W Report group spent less time completing a decision trial than in the No W Report group. If a shorter response time could be viewed as a cognitive benefit, then it seems that having attended to W might have encouraged the participants to finish the task sooner. We speculate that by raising an awareness to the decisional onset (i.e., having to attend and report W), one might have felt less of a need for further deliberation (e.g., “I have already felt the onset of a decision, therefore I should go with it.”). In contrast, participants in the No W Report group did not explicitly attend to the decision onset and therefore might spend more time deliberating, resulting in a longer response time. In this manner, it appears that by being aware of the decision onset has an influence on a subsequent action, namely shorter time spent on the decision.

Similarly, we have observed that the difficulty ratings in the W Report group was lower than the No W Report group (although this data trend did not reach statistical significance and did not pass the BH multiple comparison correction). This data trend appears to go hand in hand with the RT data such that the W Report group took less time to make a decision and also rated the stimuli as less difficult than the No W Report group. As with response time, these results add to the general view of the study that temporal experience of the decision onset has an influence (and perhaps a cognitive benefit) on a subsequent action or thought.

### Limitations and future directions

#### Time W

The current study was intended to capture the earliest subjective moment of a decision onset. Time W, as backward referenced from the response time, may appear to be less straightforward compared to a more traditional way of measuring decision time from the offset of the stimulus. Our choice of using W is because it is more informative in the context of Libet-based consciousness research. W not only conveys how early or late a decision is in the same manner as a traditional method would (see Supporting information for correlation analysis between W, traditional decision time and response time), it also conveys how much time before a final response is made. The latter is tied to Libet’s veto window and the view that free will and consciousness have a purpose.

It is also important to note that time W in our study does not necessarily represent the veridical decision time nor does it reflect the final decision, and that other activities such as additional deliberation and mind changing may occur after participants provide their subjective estimate of W. Future investigation of these decision-making components could reveal further insights into how consciousness may play a role in subsequent thoughts and behaviors.

While the primary focus of the current study is on the subjective experience of the decision time W, investigation of a veridical W remains open. In the current study, we relied on the W report in the No Tone condition as baseline. However, this baseline measure does not necessarily represent the true timing of the decisional urge as it too could be delayed or biased by subjective reports. Efforts certainly have been made to identify the veridical W [e.g., 25,26]. It would be important to see whether veridical W would also have an impact on subsequent assessment of decisional difficulty.

We employed the tone manipulation procedure to modulate the W report. The manipulation has been demonstrated here and elsewhere to effectively shift W reports or other subjective times in the direction of the tone [e.g., 15,27]. The effectiveness of the manipulation is believed to be due to the brief delay inserted between the manual response and the tone, rather than to the presence of the tone itself. In the context of our study, there could be a concern that the mere presence of the tone increased task complexity, rendering a higher difficulty rating. However, we speculate that the mere presence of the tone did not directly influence the difficulty evaluation because there was no difference in difficulty ratings in the delayed tone and no tone conditions in the group of participants who did not have to report W. These results thus support our view that the presence of the tone does not have a direct effect on difficulty assessment. However, it is possible that the presence of the tone could have an additive effect such that the presence of the tone could slow down the processing of W information (perhaps in line with the psychological refractory period), rendering a later W report and a higher difficulty rating. The additive effect should be considered in future investigations.

Another question that could be discussed is the extent to which the W report is modulated by the tone manipulation. Kang et al. demonstrated that their subjective W did not vary from the objectively measured decision time [25]. In addition, the authors also discussed the idea of a precision window. In the current study, we relied on the tone manipulation to modulate W reports. It could be speculated that the reported W in our study fluctuates within these precision boundaries. When a delayed tone is used, the tone could have modulated the reported W closer to the later boundary point, resulting in a later W report. This speculation would be interesting to explore in future experiments.

Relatedly, the current study prompts the questions of whether the tone manipulation has an impact on the veridical W itself, and whether subjective W could still be modulated when not engaged in the W report. Regarding the first question, it is speculated that the tone manipulation does not affect veridical W. Rigoni and colleagues showed that while the tone manipulation affected participants’ report of W, a scalp EEG signal (the action-effect negativity, N_AE_) was also observed, demonstrating a larger amplitude of N_AE_ in conditions with longer tone delay [28]. The ability to detect a temporal discrepancy at the neural level suggests that the veridical W is maintained within the trial. Alternatively, there is a possibility of a “history” effect whereby the tone manipulation in previous trials could influence the veridical W of subsequent trials [e.g., 29]. A Bayesian estimation approach that accounts for experience-dependent prior expectations could shed light on how tone awareness plays a role on subsequent veridical and subjective experience of W.

Another important question is whether the tone manipulation can modulate subjective W when participants are not actively engaged in reporting W. One possibility is that the tone manipulation is effective regardless of this engagement. Alternatively, it is possible that experience of W is altered only if W is being reported. If the latter, this might explain why the tone manipulation did not modulate difficulty ratings in the No W Report condition.

#### Methodology and analysis

The original Libet task of simple wrist flexion has been criticized for being an automatic action that ensues minimal consequences, and does not necessarily engage free will. The current study was designed to address some of these previous criticisms and to study a decision-making in which free will could be exercised. Thus, we chose opinion-based stimuli that had no right or wrong answer but nevertheless would result in some degree of consequences. In so doing, we recognize that the participants’ performance accuracy could not be objectively quantified as there were no correct or incorrect answers. A future investigation may consider comparing our results with other types of decision making in which performance could be objectively measured. The role of the decision time (W) could potentially vary with different types of decision tasks.

The experimental manipulation in Experiment 2 was used to bias the perception of difficulty of previously rated medium-difficulty stimuli. To best maintain the integrity of the manipulation, we could not assess for baseline ratings from the target participants; we could only assume that the focus participants had a similar perception of difficulty as the independent raters.

To our knowledge, this is the first study to examine the possible influence of reported W on subsequent thoughts. Our Interpretations of the results are based on traditional analyses as well as multiple corrections procedure and the Bayes factor analysis. While the primary findings met statistical criteria supporting the null or alternative hypotheses, Bayes factor values, as supportive evidence, were smaller, prompting a need for future replications and alternative interpretations.

#### Libet’s veto theory

Libet proposed that consciousness and free will have a purpose. The time window between decision and a motor response is assumed to be the conscious time of deliberation and cancelation. Isham et al. observed that this veto period was longer in the easy decision trials and shorter for the difficult trials [10]. Based on these previous findings and the veto theory, coupled with the current research on subjective W, a potential research direction is to examine whether manipulating W to be early or late could lead to a subjective experience of having deliberated more or less. If the perceived deliberation could be manipulated as such, this then could influence subsequent thoughts or ideas (e.g., difficulty ratings) while aiding further exploration of what Libet’s veto window represents.

#### Intentional binding

The evidence from Experiment 2 implying that difficulty rating does not influence W questions the robustness of Intentional Binding theory. According to the theory, an effortful action leads to a temporal compression between the timing of action and the ensuing effect [27]. Results from Experiment 2, however, showed that perceiving a decision to be effortful and difficult led to a time dilation rather than a time compression between W and keypress. Our results provide an instance where Intentional Binding may not apply. Future studies may wish to explore this in depth by acquiring the traditional measures used in the Intentional Binding paradigm.

#### Applications

A recent study showed that altering perceived time also influenced physiological responses; e.g., perceived time alters the blood sugar level in people with type 2 diabetes [30]. Their results provide additional support to the perspective that temporal experience plays an influential role beyond behavioral modifications. Collectively, our results and Park et al.’s observations may provide a basis for future applications that utilize time perception to better our physical, mental, and cognitive functions.

### Conclusions

In summary, the current study has provided additional insights into Libet’s W. As an index of temporal consciousness, W has been controversial. However, despite the mechanistic questions surrounding W, here we have shown that this subjective temporal experience plays a significant role in modifying subsequent thoughts and behaviors related to the assessment of task difficulty.

## Supporting information

S1 File Experiment 2 stimuli. (DOCX)

S2 File Experiment 1 data. (XLSX)

S3 File Experiment 2 data. (XLSX)

S4 File W, RT and subjective decision time (ST). ST was measured from the offset of the statement stimuli. Correlation analyses revealed ST to be highly correlated with RT and W.
(DOCX)
