# Supplementary material for: Temporal experience modifies future thoughts: Manipulation of Libet’s W influences difficulty assessment during a decision-making task
Source: PLoS One. 2020 Nov 24;15(11):e0237680. doi: 10.1371/journal.pone.0237680 (PMC7685477; doi:10.1371/journal.pone.0237680)
Supplement: S1 File — (DOCX) [file pone.0237680.s001.docx]

Experiment 2 stimuli

True Easy –15 stimuli with lowest mean difficulty rating

I would give up a friend for money.

I would give up a friend for popularity.

I am happy for the success of my friends.

I support women’s rights.

I support children’s rights.

I would go to jail for a night of fun.

I would go to jail for money.

It’s okay to drive under the influence if you feel fine.

It’s okay to drive under the influence if you are hungry.

It is fine to steal from your best friend’s cousin.

It is fine to steal from your best friend’s sibling.

I want others to be respectful to me.

I want others to worship me.

I would give up my savings to pay for my mother's hospital bills.

I believe in fate.

Set A – 15 stimuli with moderate mean difficulty rating

Money can’t buy happiness.

To save the village it is okay to sacrifice a child.

I would give up my savings to pay for my mother’s new car.

Children should not be allowed access on Facebook.

If I found money, I would keep the cash.

You should not curse in front of women.

It is okay to speed when I am running late to class.

It is okay to speed when there is a family emergency.

It is okay to have sex before marriage.

The strength of a nation is judged by the way its people are treated.

In a desperate situation, it is okay to consume a sick child for food.

In a desperate situation, it is okay to consume a dog for food.

I would give up friends for more friends.

I am happy for the success of strangers.

Bad people deserve to be punished.

Set B – 15 stimuli with moderate mean difficulty rating

I would do anything for a million dollars.

Freedom is more important than money.

It’s okay to cheat in a game.

It’s okay to publically criticize celebrities.

Children should not have cell phones.

It is my responsibility to help conserve energy.

It is my responsibility to help conserve wildlife.

If I found money, I would return it.

A cold-blooded killer should be tortured.

It is better to look forward than to look back.

It is better to build a career before marriage.

If I saw someone being attacked, I would help.

I sympathize with wealthy people who have just lost their jobs.

okay to bend the rules to help someone out.

It is fine to steal from your best friend’s enemy.

True Difficult – 15 stimuli with highest mean difficulty rating

Lying is okay if it keeps me safe.

Lying is okay if it keeps my country safe.

I am happy for the success of my enemies.

I sympathize with wealthy people who have no real friends.

If I won the lottery, I would give it all to my family.

A person’s true character is revealed through power.

A person’s true character is revealed through love.

A person’s true character is revealed through leadership.

Freedom is more important than death.

Freedom is more important than love.

I would steal to give to my children.

It’s better to forget than to forgive.

It’s better to remember pain than to forget pleasure.

It’s better to co-habitat before marriage.

I’d rather receive an A- than B+.
