# Supplementary material for: Temporal experience modifies future thoughts: Manipulation of Libet’s W influences difficulty assessment during a decision-making task
Source: PLoS One. 2020 Nov 24;15(11):e0237680. doi: 10.1371/journal.pone.0237680 (PMC7685477; doi:10.1371/journal.pone.0237680)
Supplement: S4 File — ST was measured from the offset of the statement stimuli. Correlation analyses revealed ST to be highly correlated with RT and W. (DOCX) [file pone.0237680.s004.docx]

**Correlation Analyses for W, RT, and ST**

In addition to W and RT, we have also considered the subjective decision time (ST) variable measured from the end of the stimuli. Our correlational analyses revealed that ST shares attributes with W and RT as shown below. Due to shared attributes with W and RT, ST was not considered in the primary analysis.

1. ST and W - ST and W have the same foundation, namely, the subjective feeling of a decision onset. Correlation analysis revealed a significant relationship between ST and W (*Pearson’s r =* -.449 in the Delayed Tone condition and -.498 in the No Tone condition*).* Furthermore, in the same manner as W, ST was influenced by the tone manipulation: ST was earlier (971.38 ms after the statement is presented) when no tone was presented, and later when a delayed tone was presented (1094.94 ms after the statement was presented), *t* (23) = 2.179, *p* = .040. W is influenced by the tone in a similar manner: in the No Tone condition, W preceded the manual response by 410.90 ms whereas in the Delayed Tone condition, W preceded the manual response by 371.31 ms, implying that W in the No Tone condition was earlier than W in the tone condition, *t* (23) = 2.468, *p* = .021. On this basis, W and ST share many common attributes. An important issue, however, is that ST is also related to reaction time, which W is not.

1. ST and RT. From our data, we have observed that an early decision is associated with a shorter response time, and a late decision is associated with a longer response time. This is supported by the correlation analysis revealing that ST and response time are highly correlated; Pearson’s *r* were .706 (*p* < .001) and .729 (*p* < .001), for the Delayed Tone and No Tone conditions, respectively. The results suggest that ST conveys similar information as response time. Due to ST’s shared attributes with RT, as well as with W, as described above, results surrounding ST would be somewhat challenging to interpret in the context of our experiment.

**Rationale for W**

We chose to use W in our experiment because W better served the experimental purpose for two reasons. First, W does not necessarily vary with the general response time. In some instances, an earlier decision time is not indicative of an immediate motor execution. By the same token, a late decision time does not necessarily lead to a delay in the motor execution. Thus, W and response time are not significantly associated (Pearson’s *r:* -.221, *p* = .299 and -.357, *p* = .087, in the tone and no tone condition, respectively). In this manner, we were able to dissociate W and RT, enabling us to compare how each measure predicts difficulty ratings.

In addition, we chose to focus on W, instead of ST, for both theoretical reasons and its past use in the literature. Primarily, this is because W provides additional information that is crucial to consciousness research. According to Libet, W is the temporal window in which one consciously evaluate, and possibly veto, an action-in-progress. By manipulating W with a delayed tone, we were able to influence the subjective report of W, which represents the length of this temporal window. In the current study, as well as in our previous work (Isham et al., 2017), we have observed that a larger W magnitude (longer veto window) is associated with easier decisions and a smaller W magnitude (shorter veto window) is associated with difficult decisions. These findings thus have raised the question of whether the time window serves the cancelation function as previously proposed by Libet, subsequently opening new avenues for research on the function of consciousness.
